# *Tricholoma matsutake* polysaccharides suppress excessive melanogenesis via JNK-mediated pathway: Investigation in 8- methoxypsoralen induced B16–F10 melanoma cells and clinical study

**DOI:** 10.1016/j.heliyon.2024.e29363

**Published:** 2024-04-08

**Authors:** Yang Yang, Zheng Lv, Quan An, Detian Xu, Longjie Sun, Yiming Wang, Xuexue Chen, Xue Shao, Tong Huo, Shuangrui Yang, Jiali Liu, Haoshu Luo, Qianghua Quan

**Affiliations:** a State Key Laboratory of Agrobiotechnology, College of Biological Sciences, China Agricultural University, Beijing, 100193, China; b Yunnan Baiyao Group Co., Ltd., Kunming, 650504, China; c East Asia Skin Health Research Center, Beijing, 100037, China; d Shanghai Skin Disease Hospital, Tongji University Medical School, Shanghai, 200050, China; e The Ice Dermalab, Shanghai, 200050, China; f Kunming Hospital of Traditional Chinese Medicine, Kunming, 650011, China

**Keywords:** *Tricholoma matsutake*, Polysaccharide, Melanogenesis, Hyperpigmentation, c-Jun N-Terminal kinase

## Abstract

Skin hyperpigmentation is a worldwide condition associated with augmented melanogenesis. However, conventional therapies often entail various adverse effects. Here, we explore the safety range and depigmentary effects of polysaccharides extract of *Tricholoma matsutake* (PETM) in an *in vitro* model and further evaluated its efficacy at the clinical level. An induced-melanogenesis model was established by treating B16–F10 melanoma cells with 8-methoxypsoralen (8-MOP). Effects of PETM on cell viability and melanin content were examined and compared to a commonly used depigmentary agent, α-arbutin. Expressions of key melanogenic factors and upstream signaling pathway were analysed by quantitative PCR and western blot. Moreover, a placebo-controlled clinical study involving Chinese females with skin hyperpigmentation was conducted to measure the efficacy of PETM on improving facial pigmented spots, melanin index, and individual typology angle (ITA°). Results demonstrated that PETM (up to 0.5 mg/mL) had little effect on the viability and motility of B16–F10 cells. Notably, it significantly suppressed the melanin content and expressions of key melanogenic factors induced by 8-MOP in B16–F10 melanoma cells. Western blotting results revealed that PETM inhibited melanogenesis by inactivating c-Jun N-terminal kinase (JNK), and this inhibitory role could be rescued by JNK agonist treatment. Clinical findings showed that PETM treatment resulted in a significant reduction of facial hyperpigmented spot, decreased melanin index, and improved ITA° value compared to the placebo-control group. In conclusion, these *in vitro* and clinical evidence demonstrated the safety and depigmentary efficacy of PETM, a novel polysaccharide agent. The distinct mechanism of action of PETM on melanogenic signaling pathway positions it as a promising agent for developing alternative therapies.

## 1 Introduction

Skin hyperpigmentation is a worldwide skin pigment disorder, significantly impacting the quality of life and mental well-being of patients [1,2]. Pigment spots are commonly observed on the facial and neck areas of those patients, which are considered as an aging hallmark in East Asian [[3], [4], [5]]. Conventional therapeutic options for skin hyperpigmentation disorders have yielded a number of adverse effects and varied outcomes. Widely used depigmenting agents, such as hydroquinone and azelaic acid, could cause irritation and erythema [2,6]. Energy-based therapies and chemical peeling are linked with a high risk of post-inflammatory pigmentation and relapses in coloured skin [6,7]. Therefore, safe and efficient topical agents for managing skin hyperpigmentation are urgently needed.

The pathogenesis of skin hyperpigmentation is closely associated with excessive melanogenesis [2,7]. Researches have uncovered various signaling pathways and key factors at different levels that orchestrate the regulatory network of melanin synthesis [8,9]. A series of enzymatic reactions are involved in the production of melanin, and the reaction rates of which are mainly regulated by tyrosinase, tyrosinase-related protein-1 (TRP-1), and TRP-2 [10]. Reducing the activity of these enzymes, particularly tyrosinase, is a common approach to treat hyperpigmentation [1]. Notably, the mRNA expressions of these three enzymes are modulated by microphthalmia-associated transcription factor (MITF) [8,11]. Upstream signaling pathways such as cAMP/protein kinase A (PKA), mitogen-activated protein kinase (MAPK), and phosphoinositide 3-kinase/Akt (PI3K/Akt) modulate the expression of MITF [9,12]. Thus, compounds targeting these melanogenic signaling pathways have great potential for developing effective depigmenting strategies.

Plant and fungus-derived ingredients with low or no cytotoxicity are of particular interest in developing depigmentary agents. An increasing number of plant and fungus-derived depigmentary ingredients have been reported: plant-derived arbutin and fungus-derived kojic acid have been widely used in skin lightning products [[13], [14], [15]]. However, most of these agents act as tyrosinase inhibitors, with few targeting melanogenic signaling pathways or having their mechanisms of actions thoroughly investigated [[16], [17], [18], [19]]. Moreover, the lack of safety and efficacy evaluation at the clinical level limits the practical value of such natural compounds.

*Tricholoma matsutake*, a wild edible mushroom known for its significant medicinal properties and minimal cytotoxicity, contains a plethora of bioactive compounds. These include polysaccharides, volatile substances, and steroids, which have been meticulously identified through chemical analysis and are known to confer a wide array of health benefits [20]. *T. matsutake* polysaccharides stand out due to their high anti-inflammatory, anti-oxidant, anti-tumor, and anti-photoaging activities, as evidenced by *in vitro* studies [21]. Here, we characterized a low-weight fraction of polysaccharides extract of *Tricholoma matsutake* (PETM) and evaluated its safety and bioactive potential in treating skin hyperpigmentation. *In vitro* excessive melanogenesis model and a clinical trial were utilized for examining the depigmentary activity of PETM. Further investigations into melanogenic signaling pathways unveiled the distinct mechanism through which PETM suppressed excessive melanogenesis.

## 2 Materials and methods

### 2.1 PETM preparation and formulation of test serums

PETM was extracted and purified following [22]. Briefly, the crude *T. matsutake* polysaccharides extract (Yunnan Baiyao Group Health Products Co., Ltd., China) was dissolved in distilled water and loaded onto a DEAE-FAST-FLOW cellulose column (Yuanye BioTechnology Co., Ltd., China). Samples were eluted with distilled water and a series of sodium chloride solutions (0.2 mol/L, 0.5 mol/L, and 1.0 mol/L) at a flow rate of 1 mL/min. Total carbohydrate content was determined following the phenol-sulfuric acid method. The fraction eluted with 0.2 mol/L sodium chloride was designated as PETM and was further dialyzed in distilled water (3500 Da). For molecular weight determination, the freeze-dried PETM was dissolved in the mobile phase (0.05 mol/L sodium chloride) to make a 5 mg/mL solution. An SK805-804-802 column (300 mm × 7.8 mm inner diameter, BoRui Saccharide Biotech, China) was used for the high-performance gel permeation chromatography (HPGPC) analysis. 20 μL sample was injected with a flow rate at 0.6 mL/min. The temperature of the column was set at 40 °C. Dextran standards (Yuanye BioTechnology Co., Ltd., China) were used to generate a standard curve. Purified PETM were then used for subsequently studies.

For the clinical trial, a carrier-only serum without depigmenting activity was prepared as the placebo control. The same formulation was used for preparing 1 % PETM serum, 0.5 % PETM serum, and 1 % α-arbutin serum (Supplementary Table S1). Polysaccharide content and molecular weights of PETM components were analysed with high-performance gel permeation chromatography (HPGPC, Shimadzu, China). PETM (20 μL) was injected into the column (BRT105-104-102; 8 × 300 mm; BoRui Saccharide, China). The flow rate of the mobile phase (0.05 mol/L NaCl) was set to 0.6 mL/min for elution, and the column temperature was set to 40 °C.

### 2.2 Cell viability assay

The 3-(4, 5-dimethylthiazolyl-2)-2, 5-diphenyltetrazolium bromide (MTT) cell proliferation and cytotoxicity kit (Beyotime, China) was used following the manufacturer. Briefly, cells of the B16–F10 melanoma line (CRL-6475, ATCC, China) were cultured in Dulbecco's Modified Eagle Media (DMEM) high-glucose medium (Gibco-Sigma, USA) in 96-well plates (5 × 10^3^ cells/100 μL medium) for 24 h. B16–F10 cells were treated with either 8-methoxypsoralen (0.05, 0.1, 0.15, 0.3, 0.5, and 1.0 mmol/L) for 24 h or PETM (0.1, 0.2, 0.3, 0.4, and 0.5 mg/mL) for 24 h or 48 h. 10 μL MTT (5 mg/mL) was added prior to another 4-h incubation. Formazan solubilization solution (100 μl) was then added to each well and mixed until the purple crystals were fully dissolved. Absorbance at 570 nm was measured by a microplate reader (Tecan, China). Percentages of cell viability was calculated as OD_S__ample_/OD_B__lank_ × 100. Three independent experiments were performed with six technical replicates per experiment.

### 2.3 Scratch assay

B16–F10 cells were seeded into 6-well culture plates, and were incubated at 37 °C, 5 % CO_2_ overnight. Cell layer in each well was scraped in a straight line by 200-μL pipette tips. After scratch, cells were gently washed with phosphate-buffered saline (PBS, pH 7.2) twice. For PETM treated group, 0.5 mg/mL PETM was added to the fresh medium. Images at 0, 24 and 48 h post scratch were taken at the same spot and analysed by using ImageJ.

### 2.4 Melanin content assay and tyrosinase activity assay

B16–F10 cells at a concentration of 6 × 10^5^ cells/well were incubated in 60-mm culture plates at 5 % CO_2_ 37 °C for 12 h, allowing cells to adhere. Cells were then pre-incubated with 1 mL PETM or α-arbutin dissolved in DMEM for 6 h. Cells pre-treated with the same volume of DMEM medium were used as the control. For melanin content and tyrosinase activity analysis, cells were treated with 50 μmol/L 8-MOP and cultured for another 36 h and 16 h, respectively. Cells were then harvested and were washed with PBS at pH 7.2 twice.

Melanin content and tyrosinase activity were analysed following an established protocol [23]. In brief, 1 mol/L NaOH (with 10 % DMSO) was added to the cultured cells, which were then heated in a water bath at 80 °C for a 30-min duration. Absorbance at 490 nm was measured. For tyrosinase activity measurement, B16–F10 cells were lysed with PBS containing 0.5 % Triton X-100 and 1 % sodium deoxycholate. Total protein concentration was measured with a BCA kit (CWBiotech, China). 50 μg protein sample was incubated at 37 °C with 2.0 mmol/L l-dopa for 1 h. Absorbance value at 475 nm was then measured. Tyrosinase activity was calculated as: OD_S__ample_/OD_Control_ × 100 %. Three independent experiments were performed with three technical replicates per experiment.

### 2.5 RNA extraction and quantitative PCR (qPCR) analysis

Total RNA was extracted from B16–F10 melanoma cells treated with 8-MOP for 12 h using RNAios Plus reagent (Takara, Kusatsu, Japan). RNA concentration was measured with a Nano300 ultramicro-spectrophotometer (All Sheng, China). M-MLV reverse transcriptase (Promega, China) was used to synthesize cDNA following the manufacturer's protocol. qPCR was performed by using SYBR Green on a Light Cycle® Real-Time PCR machine (Roche, USA). *GAPDH* gene was used as an internal control for normalization. Transcript levels of target genes were calculated relative to a reference gene using the *2*^–ΔΔCt^ method [24]. Primers used in this study were synthesized by Sangon Biotech (China) and are shown in Supplementary Table S2.

### 2.6 Western blot analysis

B16–F10 cells were incubated with 50 μmol/L 8-MOP for a 24-h duration, then collected and treated with protein lysis buffer (Beyotime, China). Total protein extract was obtained by centrifuging at 12000 rpm for 30 min at 4 °C. Proteins (30 μg protein/lane) isolated by SDS-PAGE were transferred to polyvinylidene fluoride membranes (Millipore-Sigma, USA). After blocking in 5 % skim milk for 1 h, membranes were incubated with the primary antibody overnight at 4 °C, then incubated with the secondary antibody for 2 h (at room temperature). Antibodies are listed in Supplementary Table S3. Western blot images were captured by a Tanon 5200 multi chemiluminescence imaging system (TANON SCIENCE & TECHONLOGY, China).

### 2.7 Clinical study and skin hyperpigmentation evaluation

Guided by epidemiological analyses of melasma prevalence in China, our clinical trial specifically targeted volunteers who are most vulnerable to this condition. Specific inclusion criteria were applied: Chinese indoor-working women aged 30–50 who had a clinical diagnosis of melasma. A minimum modified melasma area and severity index (mMASI) score was set as 5.8 for participant recruitment. The exclusion criteria prevented participation of women who had: (1) been pregnant or lactating at the time, or were considering pregnancy in the following six months; (2) used whitening or depigmentation treatments (including topical and/or systemic medicine, chemical peeling, photoelectric therapies, etc.) in the two months prior to the study; (3) taken systemic hormonal drugs or immunosuppressants within the last month; (4) a history of cosmetic contact dermatitis; (5) any facial skin disease that may have affected the diagnosis or evaluation of melasma; or (6) a serious heart, brain, liver, kidney, blood, or other systemic disease or immune deficiency. All participants gave written informed consent before the clinical trial began. Forty suitable volunteers were randomly assigned into different groups; 36 participants completed the study (the placebo group, n = 10; 0.5 % PETM serum group, n = 10; 1 % PETM serum group, n = 8; 1 % α-arbutin serum group, n = 8).

The clinical trial was conducted following randomized, double-blind, placebo-controlled rules. In brief, participants were randomly assigned to groups. Each individual applied the assigned serum (0.5 % PETM, 1 % PETM, 1 % α-arbutin, or placebo) to their face after washing with a facial cleaner every morning and evening for 16 weeks. Because UV exposure is a major causative factor for melasma [8], a sunscreen (SPF50+，PA+++) was provided and all participants applied it every morning during the study.

For each participant, facial images at different time points were taken by a VISIA-CR system (Canfield Scientific, USA) in standard light mode 2. Melanin index and ITA° at hyperpigmented spots were measured by using Cutometer® Dual MPA 580 (Courage + Khazaka electronic GmbH, Germany). Before collecting facial images, participants washed their faces with facial cleanser and waited in a temperature- and humidity-controlled room for at least 30 min. Facial images were imported into Image-Pro Plus (IPP) Media Cybernetics, USA) for further analysis. For each participant, hyperpigmented areas were measured in a selected region at the baseline and after the 16-week treatment. Relative spot area was calculated as: hyperpigmented spot area in the selected region/total area of selected region × 100 %. All skin measurements were performed in a room with controlled environmental conditions. Treatment efficacy was evaluated as the percentage of volunteers who achieved at least an 8 % reduction in the relative area of their hyperpigmented spots post-treatment.

### 2.8 Statistical analysis

All statistical analyses were performed with GraphPad PRISM7 software (GraphPad Software LLC, USA) using Student's t-test.

## 3 Results

### 3.1 Effects of PETM on the viability and migration of B16–F10 cells

While characterizing *T. matsutake* polysaccharides (TMP), we discovered an 18.6-kDa fraction that possessed relatively high anti-melanogenic activity (Supplementary Figure S1 and S2). This low-weight polysaccharide fraction was designated as polysaccharide extract of *T. matsutake* (PETM) and used in further investigation.

**Fig. 1:**
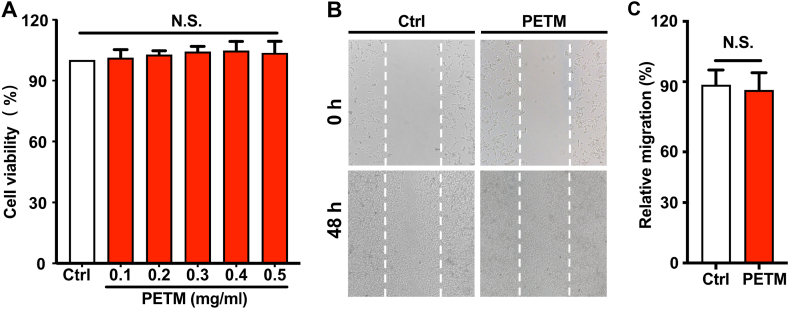
Effects of PETM on the viability and migration of B16–F10 cells. (A) Effects of different concentrations of PETM on B16–F10 cell viability. (B) Effects of 0.5 mg/mL PETM on B16–F10 cell migration. Bar = 50 μm. (C) Analysis of migration cells in the scratch assay. After 48 h, cells that migrated to the scratch area were quantified. N.S., no significant difference (Student's *t*-test, treatment vs. blank control). Data were collected from three independent experiments. Error bars show standard deviation.

To determine the safety range of PETM treatment, we firstly tested the cytotoxicity of PETM in B16–F10 melanoma cells after a 48-h incubation (Fig. 1A). The results showed that PETM (up to 0.5 mg/mL) exerted little cytotoxic effects on B16–F10 cells. Furthermore, effect of PETM on B16–F10 cell motility was also examined by using the scratch assay. The migration rate exhibited no significant difference between the group treated with 5 mg/mL PETM and the non-treated control (Fig. 1B and C). Collectively, these results demonstrated that PETM (up to 0.5 mg/mL) have little effect on the viability and mobility of B16–F10 melanoma cells.

### 3.2 PETM inhibited 8-MOP induced melanin synthesis in B16–F10 cells in a dose-dependent manner

**Fig. 2:**
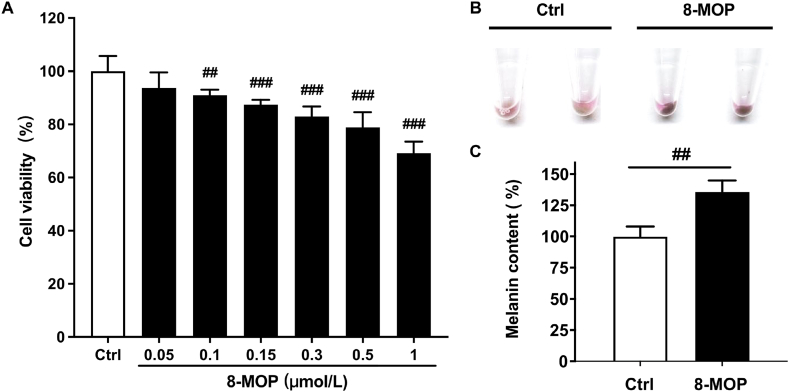
Effects of 8-MOP on the viability and melanin content of B16–F10 cells. (A) Effects of different concentrations of 8-MOP on B16–F10 cell viability. (B–C) Melanin content of B18–F10 cells with/without 8-MOP treatment after a 48-h incubation. ##*p* < 0.01, ###*p* < 0.001 (Student's t-test, model group vs. blank control). Data were collected from three independent experiments. Error bars show standard deviation.

Skin hyperpigmentation is closely associated with augmented melanin production [2,7]. Therefore, establishing an inducible melanogenesis model is crucial for screening and evaluating depigmentary compounds *in vitro*. In this study, we constructed an inducible melanin synthesis model by using a reported pigmentation enhancer, 8-MOP [25]. MTT analysis results showed that 8-MOP (up to 50 μmol/L) did not affect B16–F10 cell viability after a 48-h incubation (Fig. 2A). In addition, 50 μmol/L 8-MOP could significantly induce melanin content (Fig. 2B and C), confirming the successful development of this augmented melanogenesis model.

**Fig. 3:**
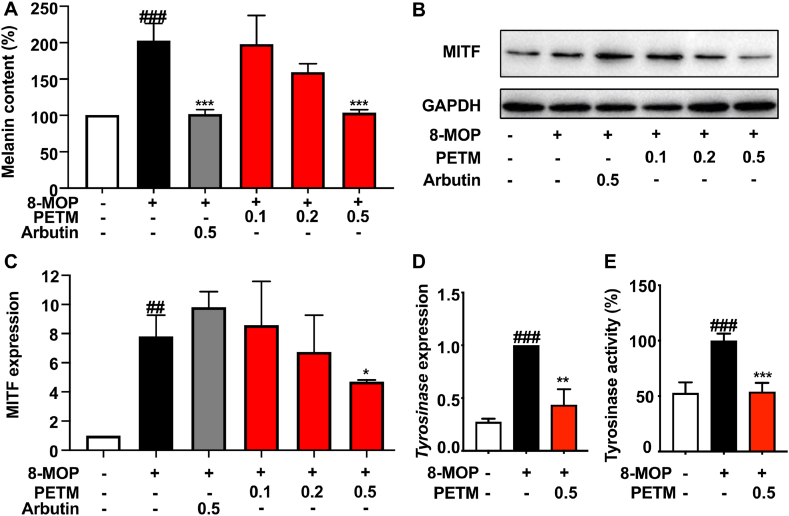
Dose-dependent effects of PETM on melanin content and MITF expression in B16–F10 cells. (A) Effects of PETM on melanin content induced by 50 μM 8-MOP. Cells were treated with various concentrations (0.1, 0.2, and 0.5 mg/mL) of PETM and 0.5 mg/mL α-arbutin, respectively. (B) Effects of PETM on the protein expression of MITF induced by 50 μM 8-MOP. Western blot was performed to analyze MITF expression. GAPDH was used as the internal control. (C) Expression levels of MITF were quantified from the western blot results. Expression levels in the control group were normalized to 1. Effects of 0.5 mg/mL PETM on the mRNA expression of *Tyrosinase* gene (D) and tyrosinase activity (E). For qPCR analysis, *GAPDH* was used as an internal control. *p < 0.05, **p < 0.01, ***p < 0.001 (Student's *t*-test, treatment vs. model group). ##*p* < 0.01, ###*p* < 0.001 (Student's *t*-test, model group vs. blank control). Data were collected from three independent experiments. Error bars show standard deviation. Microphthalmia-associated transcription factor, MITF.

To assess the depigmentary activity of PETM, melanin contents in different treatment groups were firstly measured. Alpha-arbutin (0.5 mg/mL), a well-studied skin depigmentary agent, with little cytotoxic effect on B16–F10 cells was included as the positive control (Supplemental Figure 3) [13,14]. While treatments with 0.1 and 0.2 mg/ml PETM did not yield statistically significant differences compared to the model group, a dose-dependent trend in melanin content reduction was observed (Fig. 3A). Notably, 0.5 mg/mL PETM and 0.5 mg/mL α-arbutin showed the highest inhibitory effect on melanin production induced by 8-MOP, with no significant difference in melanin content observed between these two groups. Together, these results suggested that the depigmentary activity of PETM *in vitro* was comparable to that of α-arbutin at the same concentration.

### 3.3 PETM downregulated the expression of MITF and tyrosinase activity

To investigate whether PETM functions in melanogenic signaling pathways, we next examined its effects on tyrosinase and MITF, a key transcriptional regulator of melanogenesis [8]. PETM treatment exhibited a dose-dependent decrease in both MITF and tyrosinase enzyme activity (Fig. 3 and supplemental figure 4). While no statistic difference was observed in groups treated with 0.1 and 0.2 mg/mL PETM, the 0.5 mg/mL dosage of PETM significantly reduced the expression of MITF and tyrosinase activity compared to the 8-MOP-treated group (Fig. 3B–E and supplemental figure 4). Consistent with the reduced tyrosinase activity, the mRNA expression of *Tyrosinase* was also suppressed in 0.5 mg/mL PETM-treated group (Fig. 3D). In addition, we observed no significant impact on MITF expression in α-arbutin treatment. These results suggested that PETM might reduce melanin content by targeting MITF-related signaling pathways.

### 3.4 PETM modulated the MAPK signaling pathway by inhibiting JNK activation

**Fig. 4:**
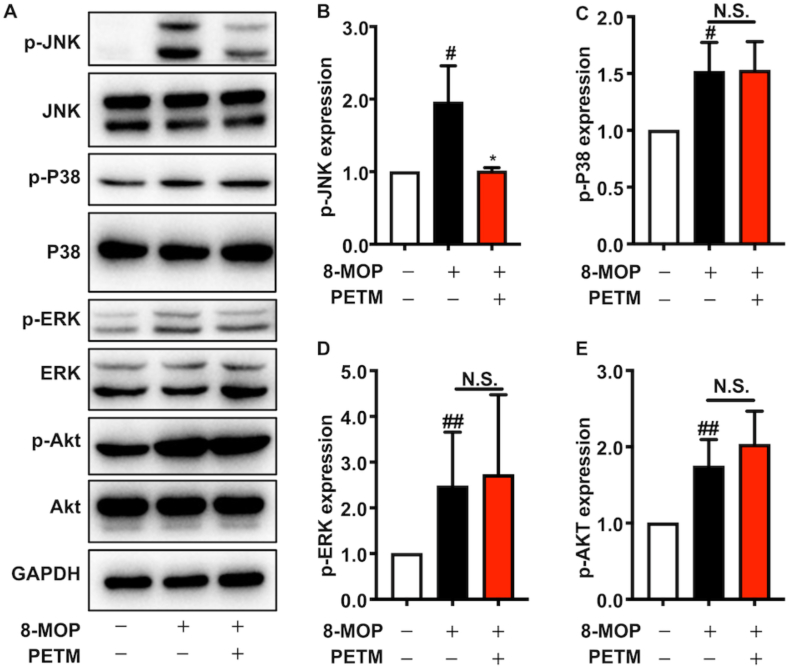
PETM mediated the phosphorylation level of JNK in B16–F10 cells. (A) The JNK, P38, ERK, Akt, phospho-JNK (*p*-JNK), p-P38, *p*-ERK, *p*-Akt levels in B16–F10 cells. GAPDH was used as the internal control. Expression levels of *p*-JNK (B), p-P38 (C), *p*-ERK (D), *p*-Akt (E) were quantified with western blot using ImageJ. Values in the blank control were normalized to 1. N.S., no significant difference, *p < 0.05 (Student's *t*-test, treatment vs. model group), #*p* < 0.05, ##*p* < 0.01 (Student's *t*-test, model group vs. blank control). Data were collected from three independent experiments. Error bars show standard deviation. C-Jun N-terminal kinase, JNK; Extracellular regulated protein kinases, ERK.

The MAPK signaling pathway plays a vital role in regulating MITF expression [9,12]. To determine whether PETM could influence melanogenic-related signaling pathways, we examined the protein expression and phosphorylation status of key proteins (JNK, P38, ERK) in the MAPK signaling pathway. Immunoblotting results showed 8-MOP induced phosphorylation levels of JNK, P38, ERK, and Akt (Fig. 4). No alteration was observed in the protein expression or phosphorylation levels of P38, ERK1/2, or AKT between the model group and PETM-treated group (Fig. 4C–E). Instead, PETM treatment significantly reduced JNK phosphorylation without affecting the protein expression of JNK (Fig. 4A and B). Together, these findings revealed that the PETM might mediate melanogenesis through inactivating the JNK pathway.

### 3.5 A JNK agonist rescued the downregulated melanin synthesis caused by PETM treatment

**Fig. 5:**
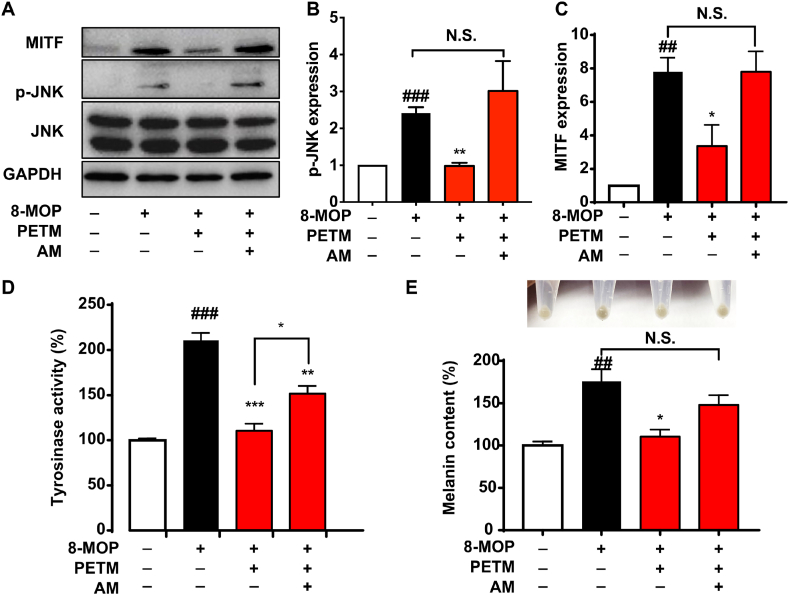
A JNK agonist abolishes the inhibitory role of PETM on melanogenesis. (A) B16–F10 cells were treated with 20 nM JNK agonist (anisomycin, AM) and PETM. Western blot was performed to determine MITF, JNK, and phospho-JNK (*p*-JNK) expression. GAPDH was used as the internal control. (B) Effects of AM on *p*-JNK expression were quantified from the western blot image. (C)–(E) Effects of AM on melanogenesis and melanin content inhibited by PETM treatment. (C) MITF expression was quantified from the western blot image. Results of relative levels of tyrosinase activity (D) and melanin content (E). Values in the blank control were normalized to 1 or 100 %. N.S., no significant difference, *p < 0.05, **p < 0.01, ***p < 0.001 (Student's *t*-test, treatment vs. model group). ##*p* < 0.01, ###*p* < 0.001 (Student's *t*-test, treatment vs. blank control). Data were collected from three independent experiments. Error bars show standard deviation. Anisomycin, AM; C-Jun N-terminal kinase, JNK; Phospho-JNK, *p*-JNK; Microphthalmia-associated transcription factor, MITF.

To confirm whether the inhibition of JNK activation underlies the suppression of melanin synthesis by PETM, we recruited a JNK agonist, anisomycin [26,27], to the PETM-treated group and assessed melanogenesis status. Immunoblotting analysis revealed that anisomycin at a dose of 20 nmol/L reversed the inhibitory effect of PETM on JNK phosphorylation (Fig. 5A and B). Thus, this dose of anisomycin was selected for further evaluation. Anisomycin abolished the inhibitory effect of PETM on the expression of MITF (Fig. 5A and C). Moreover, the tyrosinase activity and melanin content were both increased in the anisomycin-treated group (Fig. 5D and E). Together, JNK agonist could abolish the suppressive effect of PETM on JNK activation and melanin synthesis, supporting the hypothesis that PETM modulates melanogenesis through the JNK pathway.

### 3.6 PTEM showed depigmentary efficacy on human facial skin

**Fig. 6:**
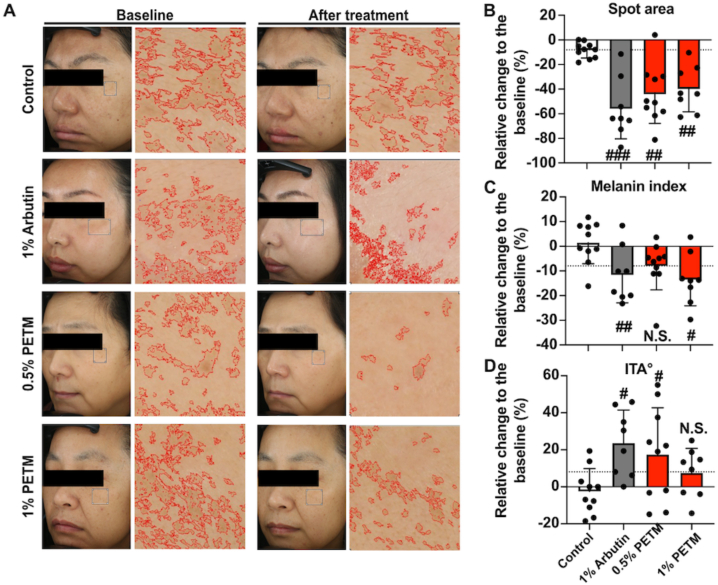
Assessment of hyperpigmented areas before and after topical treatments. (A) Facial images obtained at the baseline and after treatment with zoomed-in panels showing hyperpigmented regions. Facial images were captured using VISIA-CR. Image-Pro Plus software was used for quantification. (B–D) Changes in the spot areas (B), melanin index (C), and ITA° (D) after treatment. Changes were relative to the baseline. Dotted lines represent the relative change to the baseline at 8 %. N.S., no significant difference, #*p* < 0.05, ##*p* < 0.01, ###*p* < 0.001 (Student's *t*-test, treatment vs. blank control). Error bars show standard deviation. individual type angle, ITA°.

To evaluate the efficacy of PETM in treating skin hyperpigmentation, a randomized, placebo-controlled clinical trial was conducted with Chinese women diagnosed with melasma. The safety of 0.5 % and 1 % PETM concentrations used in this clinical trial has been assessed through animal experiments [28] and patch tests (Supplemental table S4). No serious adverse events were reported in any group during this clinical test. After a 16-week topical treatment, the hyperpigmented areas were reduced by 8.4 %, 56.0 %, 44.3 %, and 39.9 % in the placebo control, 1 % α-arbutin, 0.5 % PETM, and 1 % PETM-treated groups, respectively, compared to the baseline (Fig. 6A and B). The treatment efficacy was also evaluated based on the percentage of volunteers who achieved at least an 8 % reduction in the hyperpigmented spots area post-treatment. The 1 % PETM-treated group exhibited the same efficacy (100 %) as the 1 % α-arbutin-treated group, while the 0.5 % PETM treatment showed slightly weakened efficacy (90 %), and the placebo group reported a much lower efficacy (40 %). These results indicated that 1 % PETM treatment exhibit great response rate within the cohort.

Furthermore, the depigmentary activities in all groups were examined by quantifying two additional important parameters in the pigmented regions: the melanin index and individual type angle (ITA°). The melanin index in the placebo control group showed little change after the treatment, whereas the melanin index in 1 % α-arbutin, 0.5 % PETM, and 1 % PETM treated groups were reduced by 11.7 %, 4.9 %, and 13.5 %, respectively (Fig. 6C). ITA° values were also analysed between the baseline and after-treatment. Compared with the placebo group, ITA° values in 1 % α-arbutin, 0.5 % PETM, and 1 % PETM treatment were increased by 23.7 %, 17.4 %, and 7.5 %. 1 % PETM and 1 % α-arbutin showed similar efficacy in improving melanin index and ITA° (Fig. 6D). Thus, our results revealed the depigmentary potential of PETM at the clinical level.

## 4 Discussion

Skin hyperpigmentation is a worldwide skin condition for which the current therapies are still inadequate. Here, we evaluated the safety and bioactivity of PETM both *in vitro* and at the clinical level. Our results demonstrated that the depigmentary activity of PETM was comparable with α-arbutin. Intriguingly, this low-weight polysaccharide fraction exerted its distinct inhibitory mechanism on melanogenesis through modulating JNK activation.

Excessive melanin synthesis is considered as a major cause of hyperpigmentation [2,7]. However, it is noteworthy that many studies evaluating the depigmentary activities of candidate compounds predominantly rely on *in vitro* assays without incorporating hyperpigmented models. To address this gap and assess the depigmentary efficacy of PETM in a relevant context, we established an induced melanin synthesis model by treating B16–F10 cells with a pigmentation inducer, 8-MOP. It was reported that the UV-induced melanin synthesis model led to the accumulation of excessive reactive oxygen species (ROS) [29]. In contrast, 8-MOP-stimulated cells showed no prominent ROS effect, making it advantageous for assessing depigmentation without interference from oxidative stress [29]. In the exploration of the optimal concentration of 8-MOP treatment, we found that 8-MOP at 50 μmol/L significantly could significantly promote melanin production without cytotoxicity after a 48-h incubation (Fig. 2). Therefore, 8-MOP induced cell model can be considered suitable for future assessments in this field.

Considering the reported adverse effects associated with current therapies, it was vital to assess the safety and efficacy of PETM in this study. PETM (up to 0.5 mg/mL) did not influence the viability and migration of B16–F10 melanoma cells (Fig. 1), suggesting that PETM at this dose could not influence cell dynamics. Moreover, in the clinical study, no adverse effects were reported in the 0.5 % and 1 % PETM-treated groups, further supporting the safety of PETM treatment. To evaluate the depigmentary efficacy of PETM, α-arbutin was recruited in our study as a positive control. Alpha-arbutin is synthesized from hydroquinone or plant-derived β-arbutin, of which the depigmenting activity is about ten times higher than β-arbutin [13,30,31]. The depigmenting activity of PETM at non-cytotoxic concentrations was found to be comparable with that of α-arbutin at the same dose *in vitro*. Similarly, 1 % PETM serum demonstrated comparable efficacy to 1 % α-arbutin serum in reducing spot area, melanin index, and improving ITA° values (Fig. 6). These findings further demonstrates that PETM is a promising topical agent in treating hyperpigmentation.

Effective management of skin hyperpigmentation disorders often integrates therapies targeting different components of the pathogenesis to achieve improved therapeutic outcomes [32,33]. Therefore, it is crucial to understand the mechanism of action of PETM. Previous studies revealed that α-arbutin inhibited tyrosinase enzyme activity without influencing its mRNA expression [13,14]. Consistent with previous reports, we found that α-arbutin did not affect the expression of MITF, a key regulator of *Tyrosinase* gene expression (Fig. 3B and C). In contrast, PETM was shown to suppress the expression of MITF, resulting in reduced mRNA expression and enzymatic activity of tyrosinase (Fig. 3D and E). MITF is known to regulate the gene expression of melanogenic enzymes (such as TRP-1 and TRP-2) and crucial receptors for melanocyte function (such as the human melanocortin-1 receptor) [10,34]. Beyond their roles in catalyzing melanin synthesis, TRP proteins also contribute to melanin formation in other ways. For instance, TRP-1 is implicated in the stabilization of tyrosinase [35]. Intriguingly, TRP-2 expression has been shown to inversely correlate with melanogenesis in UVB-induced models, where it also helps protect melanoma cells from apoptosis [36,37]. It would be worthwhile to explore in future research how these melanogenic proteins regulate melanogenesis and melanocyte dynamics in an 8-MOP-induced model, as well as to further investigate the impact of PETM on these proteins and downstream phenotypes. These evidence supports that PETM could regulate MITF-related melanogenic pathways, resulting in a decrease in melanin content.

Phenolic compounds, flavonoids, and terpenoids are the major categories that transcriptionally regulate melanogenesis [[38], [39], [40], [41]]. Recent studies have highlighted that polysaccharide compounds also modulate melanogenesis at the transcriptional level. MAPK and PI3K-Akt signaling pathways are related to the depigmentary mechanism of polysaccharide compounds. Polysaccharides derived from another mushroom, *Ganoderma lucidum*, were reported to reduce the expression of *Mitf* and *Tyrosinase* in a UV-stimulated B16–F10 model via modulating MAPK and cAMP/PKA signaling pathways [42]. Polysaccharides obtained from *Stichopus japonicus* interfered with ERK activation, further suppressing MITF expression *in vitro* [43]. In addition, polysaccharide extract from *Leonurus japonicus* shows anti-tyrosinase activities in an oxidative-stressed model, and modulates MITF via PI3K-Akt and β-catenin pathway [44].

Distinctly, we found that PETM treatment inhibited melanogenesis through mediating JNK activation (Fig. 4). The involvement of JNK in melanogenesis has been unveiled in previous studies: It was reported that psoralen derivatives like 8-MOP and 5-methoxypsoralen stimulate melanin production by promoting the phosphorylation of JNK, P38, and Akt [45]. A flavonoid compound promoted melanogenesis by upregulating the phosphorylation of JNK and P38 [46]. In addition, adding 10 μM JNK inhibitor II significantly decreased melanin content in an α-melanocyte-stimulating hormone-induced hyperpigmentation cell model [47]. These results highlight the vital role of JNK in melanogenesis. Notably, the role of JNK in melanogenesis is associated with the stimuli that induce melanin synthesis. Consistent with previous report, the upregulated phosphorylation level of JNK, P38, and Akt was detected in 8-MOP treated groups in our study (Fig. 4). Adding a JNK agonist abolished the inhibitory effect of PETM on MITF-mediated melanogenesis (Fig. 5), which further confirmed that PETM regulates JNK activation. However, anisomycin only partially rescued the tyrosinase activity and melanin content that were inhibited by PETM. It has been reported that melanin synthesis could be regulated via MITF-independent pathways and epigenetic pathways [48,49]. PETM might also function through these alternative melanogenic mechanisms, which need further investigations. Overall, our results demonstrate the key role of JNK activation in PETM-mediated attenuation of melanogenesis.

In conclusion, our study identifies PETM as a novel depigmentary agent, effectively reducing melanogenesis through the suppression of JNK phosphorylation. Although we demonstrate that this novel polysaccharide extract exhibits depigmentary activity both *in vitro* and *in vivo*, the clinical trial has involved a relatively small number of subjects. Large-scale and multicentre clinical trial could be focused in future studies. Conducting clinical validation to thoroughly evaluate the impact of PETM on key enzymes and melanogenic factors is of significant value as well. The insights gained from this study will aid on the development and application of PETM-based treatments as safe and efficacy alternatives for skin hyperpigmentation.

## Ethical approval and consent to participate

The study was conducted in accordance with the Declaration of Helsinki, and approved by Ethics Committee on Biomedical Research, West China Hospital of Sichuan University (No.1211/2021). This clinical trial was pre-registered at Chinese Clinical Trial Registry (date of registration: 2021-12-11). All participants gave written informed consent before the trial began.

## Funding

This research was funded by Yunnan Science and technology project (grant number 2018ZF005).

## Data availability statement

All data generated during this study are included in this article and its supplementary information files.

## CRediT authorship contribution statement

**Yang Yang:** Writing – original draft, Formal analysis, Conceptualization. **Zheng Lv:** Writing – original draft, Investigation, Conceptualization. **Quan An:** Writing – review & editing, Conceptualization. **Detian Xu:** Writing – original draft. **Longjie Sun:** Investigation. **Yiming Wang:** Formal analysis, Conceptualization. **Xuexue Chen:** Investigation. **Xue Shao:** Resources. **Tong Huo:** Resources. **Shuangrui Yang:** Investigation. **Jiali Liu:** Writing – review & editing, Conceptualization. **Haoshu Luo:** Writing – review & editing, Conceptualization. **Qianghua Quan:** Writing – review & editing, Formal analysis, Conceptualization.

## Declaration of competing interest

The authors declare the following financial interests/personal relationships which may be considered as potential competing interests:Quan An reports financial support was provided by 10.13039/501100008871Yunnan Provincial Science and Technology Department, China. Yang Yang, Quan An, Xue Shao, Tong Huo, and Qianghua Quan are employed by Yunnan Baiyao Group Co., Ltd. If there are other authors, they declare that they have no known competing financial interests or personal relationships that could have appeared to influence the work reported in this paper.
