# Supplementary material for: Tricholoma matsutake polysaccharides suppress excessive melanogenesis via JNK-mediated pathway: Investigation in 8- methoxypsoralen induced B16–F10 melanoma cells and clinical study
Source: Heliyon. 2024 Apr 8;10(8):e29363. doi: 10.1016/j.heliyon.2024.e29363 (PMC11033116; doi:10.1016/j.heliyon.2024.e29363)
Supplement: Multimedia component 1 [file mmc1.docx]

**Supplemental materials**

**Supplemental Table S1 Formula of test serum**

| International Nomenclature Cosmetic Ingredient | % |
| --- | --- |
| 1, 3 - propanediol | 5 |
| Cyclopentadimethylsiloxane and cyclohexasiloxane | 3 |
| Gycerinum | 3 |
| 1, 2-hexanediol | 0.98 |
| PEG-20 methyl glucoside hemistearate ester | 0.6 |
| p-hydroxyacetophenone | 0.5 |
| Ammonium acryloyl dimethyl taurine/P copolymer | 0.4 |
| Methyl glucoside hemistearate ester | 0.4 |
| Ammonium acryloyl dimethyl taurine / Behenyl alcohol, polyether -25 methacrylate, cross-linked polymer | 0.25 |
| Xanthan gum | 0.1 |
| Ethylene Diamine Tetraacetic Acid | 0.05 |
| Sample | as designed |
| Water | up to 100% |

**Supplemental Table S2 Primers used in this study**

| *Mitf* | F | GCTGGAGATGCAGGCTAGAG |
| --- | --- | --- |
|  | R | GAGAGGGCATCGTCCATCAG |
| *Tyr* | F | \| CCCAGAAGCCAATGCACCTA \| \| --- \| |
|  | R | ATAACAGCTCCCACCAGTGC |
| *Gapdh* | F | ТССТССАССАССААСТССТТАG |
|  | R | ATGACCTTGCCCACAGCCTTG |

**Supplemental Table S3 Antibodies used in this study**

| \| Name \| Company \| Cat. No \| Dilution \| \| --- \| --- \| --- \| --- \| \| MITF \| Abcam \| ab140606 \| 1:1000 \| \| P38 \| CST \| 8690 \| 1:1000 \| \| JNK \| CST \| 9252 \| 1:1000 \| \| ERK \| CST \| 4695 \| 1:1000 \| \| p-p38 \| CST \| 4511 \| 1:1000 \| \| p-JNK \| CST \| 4668 \| 1:1000 \| \| p-ERK \| CST \| 4370 \| 1:1000 \| \| AKT \| CST \| 4691s \| 1:1000 \| \| p-AKT \| CST \| 9275s \| 1:1000 \| \| GAPDH \| CST \| 60004-1-Ig \| 1:10000 \| \| GAR-HRP \| Bio-Rad \| #1705046 \| 1:8000 \| \| GAM-HRP \| Bio-Rad \| #1705047 \| 1:8000 \| |
| --- | --- | --- | --- | --- | --- | --- | --- | --- | --- | --- | --- | --- | --- | --- | --- | --- | --- | --- | --- | --- | --- | --- | --- | --- | --- | --- | --- | --- | --- | --- | --- | --- | --- | --- | --- | --- | --- | --- | --- | --- | --- | --- | --- | --- | --- | --- | --- | --- | --- | --- | --- | --- |
|  |

**Supplemental Table S4 Patch test results of PETM**

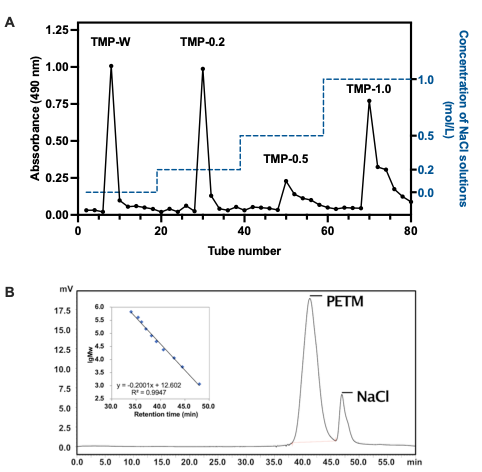


**Supplementary Figure 1.** Purification of major fractions of *Tricholoma matsutake* polysaccharides (TMP). (A) The elution profile of the crude TMP. (B) The HPGPC profile of PETM (TMP-0.2). TMP-W: fraction eluted with distilled water; TMP-0.2: fraction eluted with 0.2 M NaCl; TMP-0.5: fraction eluted with 0.5 M NaCl; TMP-1.0: fraction eluted with 1.0 M NaCl.

**Supplementary Figure 2.** Effects of major fractions of TMP on the viability and melanin content of B16-F10 melanoma cells. (A-C) Effects of major fractions of TMP (TMP-W, TMP-0.5, TMP-1.0) on melanin content. (D) Effects of major fractions of TMP on melanin content of B16-F10 cells. ****p* < 0.001 (Student’s *t*-test, treatment vs. blank control). Data were collected from three independent experiments. Error bars show standard deviation.


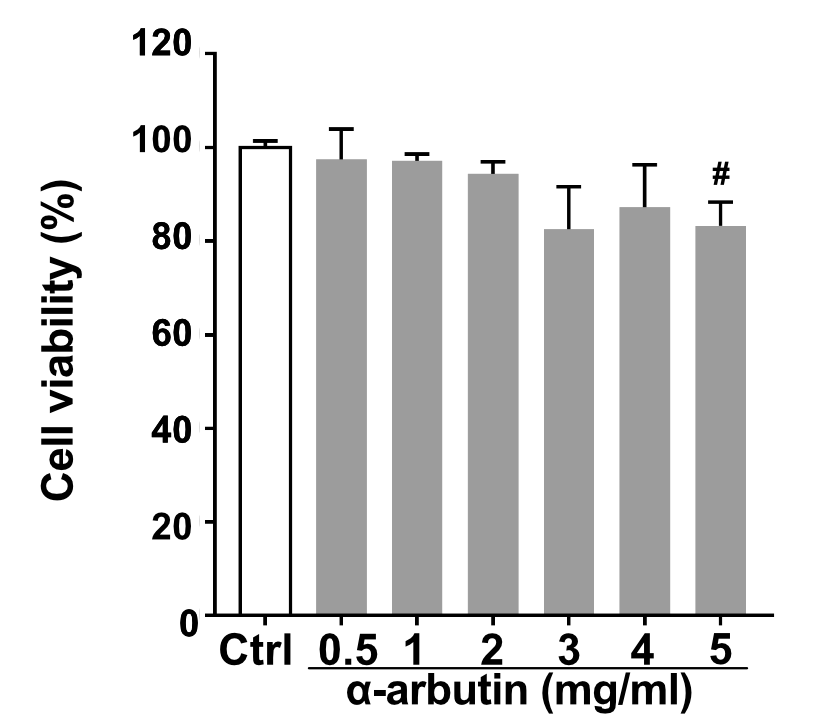


**Supplementary Figure 3.** Effects of α-arbutin on the viability of B16-F10 cells. #, *p* < 0.05, (Student’s *t*-test, treatment vs. blank control). Data were collected from three independent experiments. Error bars show standard deviation.

**Supplementary Figure 4.** Effect of low-dose PETM on tyrosinase activity. ****p* < 0.001 (Student’s *t*-test, control vs. model group); ns, no significant difference. Data were collected from three independent experiments. Error bars show standard deviation.
